# Categorisation of Colour Terms Using New Validation Tools: A Case Study and Implications

**DOI:** 10.1177/2041669518760043

**Published:** 2018-03-13

**Authors:** Shabnam Arbab, Jonathan A. Brindle, Barbara S. Matusiak, Christian A. Klöckner

**Affiliations:** Department of Architecture and Technology, Norwegian University of Science and Technology, Trondheim, Norway; Quantitative Lexicology and Variational Linguistics, KU Leuven, Belgium; Department of Architecture and Technology, Norwegian University of Science and Technology, Trondheim, Norway; Department of Psychology, Norwegian University of Science and Technology, Trondheim, Norway

**Keywords:** Colour naming, focal task, Natural Colour System, Color-aid Corporation, Chakali, colour categorisation

## Abstract

This article elaborates on the results of a field experiment conducted among speakers of the Chakali language, spoken in northern Ghana. In the original study, the Color-aid Corporation Chart was used to perform the focal task in which consultants were asked to point at a single colour tile on the chart. However, data from the focal task could not be analysed since the Color-aid tiles had not yet been converted into numerical values set forth by the Commission internationale de l’éclairage (CIE). In this study, the full set of 314 Color-aid tiles were measured for chromaticity and converted into the CIE values at the Daylight Laboratory of the Norwegian University of Science and Technology. This article presents the conversion methodology and makes the results of the measurements, which are available in the Online Appendix. We argue that some visual-perception terms cannot be reliably ascribed to colour categories established by the Color-aid Corporation. This suggests that the ideophonic expressions in the dataset do not denote ‘colours’, as categorised in the Color-aid system, as it was impossible to average the consultants’ data into a CIE chromaticity diagram, illustrate the phenomena on the Natural Colour System (NCS) Circle and Triangle diagrams, and conduct a statistical analysis. One of the implications of this study is that a line between a visual-perception term and a colour term could be systematically established using a method with predefined categorical thresholds.

## Introduction

In studies on colour categorisation, the focal task (i.e., the exemplary task) is an experiment that aims at gathering the best examples for an array of colours and calculating the loci of the elicited terms. When a claim is made that a certain language has N colour terms, the focal task examines the behaviours of a representative sample of native speakers in identifying the regions in which each of the colour terms are clustered. The results may clarify central and unusual properties of colour-term denotations. On one hand, the focal task can (dis)prove the hypothesis claiming that colour expressions are universally based on ‘favoured precepts selected from restricted regions of the colour space’ (Regier, Kay, & Cook, 2005). In such studies, the most common colour array is the Munsell colour system (Munsell, 1912). On the other hand, from a lexico-semantic perspective, experiments testing focal colour distribution can assist studies on expressions that have a diffuse set of colour denotations and provide evidence for purported ‘colour’ terms that have almost no central concentrations in a colour array, allowing for reflective assessments of the structuring of sensory meanings and the cultural component through which objects obtain their symbolic meanings (Barthes, 1967).

This study builds on the previously published dataset by Brindle (2016), which is presented in more detail in the section titled ‘Colour categorisation in Chakali’. This original study left one important question open: Do all purported ‘colour’ terms have a central concentration in a colour array? We aim to address this question by developing a mapping methodology while keeping in mind two distinct challenges in colour studies: the first being a practical and methodological one related to consistency in mapping different colour systems and the other being an ontological one regarding the status of ideophones as colour terms.

The main objectives of this study are to develop a method that brings together all the colour notations and to use the Commission internationale de l’éclairage (CIE) numerical values for the tiles in the Color-aid Corporation Chart to systematically (dis)qualify ideophonic expressions as colour terms.

### Colour Mapping Between Colour Systems

While the Munsell colour system has been widely used across disciplines such as anthropology, psychology and linguistics, various studies used the Ostwald colour system instead (Ostwald, Ostwald, & Streller, 1939); the most representative of these are a series of descriptive studies regarding the linguistic influences on colour and categorical perceptions carried out at the University of Surrey (i.e., Hippisley, Davies, & Corbett, 2008; I. Davies & Corbett, 1994; I. R. L. Davies & Corbett, 1995; I. Davies, Corbett, & Margalef, 1995; I. Davies, Jover, Vega, & Ponte, 2001; I. R. L. Davies et al., 1992). The languages studied by Surrey groups are Russian, English, Sorbian, Tsakhur, Setswana, Catalan, and Damara. The Ostwald colour system was implemented by the Color-aid Corporation in the ordered set of colour tiles in which the main features of colour are defined as hue, tint, and shade (Color-Aid-Corporation, Revised 2006). In addition, the series of studies at the University of Surrey identified the loci of the colour terms (from several languages throughout the world) within the CIE chromaticity diagram and converted some of the Color-aid codes (i.e., the tile ID numbers) into the CIE coordinates; however, the data were limited to 65 preselected tiles.^1^

Until now, no CIE conversion has ever been offered for the complete range of 314 colours in the Color-aid Corporation Chart. In addition, colour measurements presented in previous studies are not complete or, at least, were not presented as such. Photometrical data collected from all 314 Color-aid tiles under stable laboratory condition and made accessible to the research community would be a valuable contribution to the literature and to researchers of various scientific disciplines. In addition to the CIE (*u′, v*′) chromaticity graph, a graphical presentation of the Natural Colour System (NCS) was used to represent the results in a way that would be easier to interpret.

### Ontological Challenge

While the primary goal of a focal task is to identify the region in which a colour term is most appropriately located, another of its more rarely mentioned goals is to shed light on ‘secondary’ terms (i.e., those that are relatively less frequent and lack agreement among consultants as to what they denote). In this regard, the result of focal task may clarify the unusual properties of colour-term denotations, thereby providing an explanation for how a purported ‘colour’ term has almost no central concentrations in a colour array. For example, could this issue be a consequence of the a priori categorisation in the colour array presented to the consultants?

To delve deeper into this line of research, this article reports and analyses data from a field experiment conducted among native speakers of Chakali, a South-western Grusi language spoken in northern Ghana. The primary objective is to attempt to devise how to study expressions that, at first, appear to describe colours but are not yet assigned to any specific region in a colour array; in other words, this article explores how to draw a line between a visual-perception term and a colour term. To address this objective, we used a statistical analysis method to identify the dissimilarities between the terms assigned by the consultants to each colour category, the results of which are shown in the section titled ‘Statistical analysis’.

## Colour Categorisation in Chakali

This article makes use of data from Brindle (2016), who analysed colour categorisation in Chakali in an attempt to identify its colour terms and other expressions of visual perception. Brindle used a tile-naming task, a focal task and a folk-definition task, describing how native speakers (language) make use of colour terms in a folk categorization. These experiments were carried out in March 2008 and April 2013. The findings suggest that the language has at least three terms corresponding largely to white, black and red, as well as a few other terms in a ‘statistical transitional’ stage (Brindle, 2016).

Results from the tile-naming task included a series of expressions from which basic grouping, based on dominance and specificity, was determined.^2^ The triad of ‘white’, ‘black’ and ‘red’ achieved the highest consensus among the consultants, followed by terms corresponding to ‘yellow’ and ‘blue-green’ (grue). The ideophonic expression *hɔlahɔla* (and its variant: *hɔhɔla*) had a relatively high frequency, similar to the latter two colour terms, yet no significant consensus could be detected as to which colour region this term denotes. After analysing the results of the tile-naming task, many questions were raised; one was related to the need to account for *hɔlahɔla*, that is: What status is to be given to a relatively frequent term lacking dominance? Another question encompassed the larger domain of inquiry, one related to the meanings of various ideophonic terms that are infrequent and lacking dominance for the most part, yet employed by most of the consultants.

To answer these questions, we must first decipher the nature of these ideophonic expressions. Due to the extensive research conducted within the last decade,^3^ ideophones are better known and are more reliably defined. According to Dingemanse (2012), ideophones are marked words that depict sensory imagery and are connected to extralinguistic concepts: they can evoke sounds, taste, visual effects, texture, smell and so on; they often have a reduplicative structure; they are often form a separate grammatical category; and they are mainly found in Asian, African and Amerindian languages. Ideophones subsumes onomatopoeic expressions since the latter phonetically imitate the source of the sounds they describe. More generally, ideophones evoke a vivid impression of an idea.^4^ It comes as no surprise, therefore, that ideophones emerged in an experiment on expressions describing the visual appearance domain; however, it appears that ideophones have a different meaning than the one ascribed by Dingemanse, at least regarding the association between a colour region and a term.

Since the measure of dominance within the tile-naming task is intimately related to the consultants’ general concord on a colour term and its corresponding region, the focal task was considered an appropriate follow-up experiment. This experiment is designed to reveal the exemplary colour space for a set of expressions; that is, the focal task determines how the consultants treat expressions denoted by a diffuse set of tiles in the tile-naming task, thus determining whether all purported ‘colour’ terms have a central concentration in a colour array.

**Figure 1.**
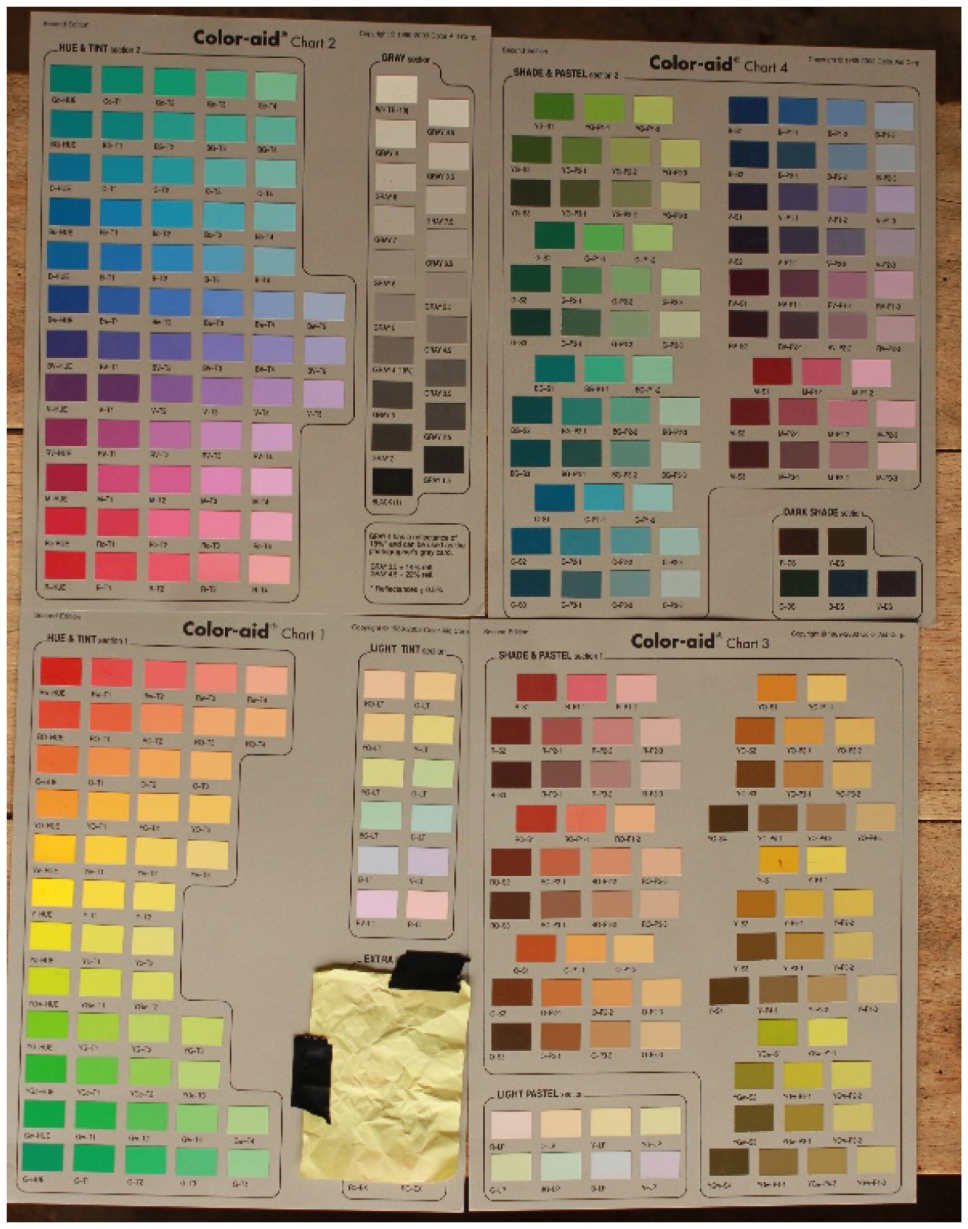
The Color-aid Charts.

Through the focal task, Brindle (2016) aimed at gathering the best examples in an array of colours. Data were collected using a relatively simple, inexpensive and replicable method consisting of laying out the Color-aid Charts on a one-meter-high table. Twenty-three adult consultants (10 men and 13 women, with the age ranging from 30 to 70 years with an average age of 45 years) stood by the table and viewed the array, displayed in Figure 1.

The experiment took place outside, under the shade of thatched roofs, between 10:00 and 14:00 over two successive, sunny days. A typical session lasted 15 minutes and all sessions were recorded and transcribed onsite. The consultants were asked, one-by-one, the question in the following diagram (Q), which was uttered by a field assistant who is a native speaker of Chakali. In each session, the colour word in the question was replaced by every stimulus expression listed in Table 2 in either alphabetical or reverse alphabetical order, which was chosen at random.

Q: kÍnà háŋ nÌ, áwèenga kā jàà ásÌàmá tÌntÌŋ?

**Table 2.** Results From the Focal Task and the Corresponding NCS Codes and CIE Coordinates (2° Observer).

Term	Color-aid		Number of answers		NCS code			*x*		*y*	*u*′		*v*′	Luminance (cd/m^2^)
búmmò	Black 1		19		NCS S 8500-N			0.32		0.3255	0.2043		0.4675	90.29
	B-S1		2		NCS S 4050-R90B			0.1949		0.2255	0.1467		0.3818	285.8
	GRAY 1.5		1		NCS S 8000-N			0.3147		0.3223	0.2018		0.465	175
	V-S2		1		NCS S 6020-R60B			0.2767		0.2406	0.2075		0.406	183.3
sÌàmá	Rw-HUE		14		NCS S 0580-Y90R			0.583		0.309	0.4208		0.5018	607.3
	Rw-T1		4		NCS S 0570-Y90R			0.5547		0.2982	0.4058		0.4907	694.1
	R-HUE		3		NCS S 1575-R10B			0.5544		0.2886	0.4142		0.4851	542.3
	Rc-HUE		2		NCS S 2060-R10B			0.5181		0.2668	0.4013		0.4648	527.8
pƱ̀mmá	WHITE 10		16		NCS S 0500-N			0.3187		0.329	0.2021		0.4692	2139
	GRAY 9.5		2		NCS S 1002-Y50R			0.3249		0.3318	0.2053		0.4716	1841
	GRAY 9		2		NCS S 1502-Y50R			0.3221		0.3298	0.2041		0.4702	1640
	GRAY 8		1		NCS S 2500-N			0.318		0.3261	0.2026		0.4676	1282
	B-LP		1		NCS S 2010-R90B			0.2958		0.3135	0.1917		0.4572	1439
	B-LT		1		NCS S 1020-R90B			0.2794		0.3089	0.1818		0.4522	1614
sƱ́sáƱ̀	Y-HUE		7		NCS S 0580-Y			0.472		0.4445	0.2555		0.5413	1541
	Yw-HUE		3		NCS S 0580-Y20R			0.5194		0.4026	0.3059		0.5334	1200
	Y-S1		2		NCS S 3060-Y			0.4627		0.4205	0.2599		0.5315	790.5
	Y-P1-1		1		NCS S 1040-Y			0.4079		0.4121	0.2288		0.5202	1503
	Yc-T1		1		NCS S 0550-G80Y			0.3957		0.4453	0.2096		0.5307	1910
	Yc-T2		1		NCS S 0540-G80Y			0.3846		0.4218	0.211		0.5206	1981
	YGw-T1		1		NCS S 0550-G60Y			0.3597		0.4531	0.1864		0.5284	1778
	YGw-T2		1		NCS S 0540-G60Y			0.3625		0.4267	0.196		0.5193	1928
	YGc-T1		1		NCS S 0565-G10Y			0.2661		0.4769	0.13		0.524	1223
	YO-P4-1		1		NCS S 6010-Y30R			0.3858		0.3574	0.2368		0.4936	350.7
	O-T3		1		NCS S 0540-Y40R			0.4527		0.3569	0.2839		0.5037	1266
	Rw-T1		1		NCS S 0570-Y90R			0.5547		0.2982	0.4058		0.4907	694.1
	RO-T1		1		NCS S 0570-Y70R			0.5557		0.3248	0.3841		0.5052	810.1
	RO-T4		1		NCS S 0540-Y50R			0.4487		0.3421	0.2891		0.496	1240
sƱ̀ɔ̀sɛ́nÍÍ	G-S1		4		NCS S 4040-B90G			0.2326		0.4169	0.1235		0.4978	451.7
	Gc-HUE		3		NCS S 3050-B60G			0.1958		0.3804	0.1092		0.4773	572.4
	YG-S1		3		NCS S 4050-G30Y			0.2965		0.4815	0.1449		0.5295	475.8
	YG-T1		2		NCS S 0560-G20Y			0.3048		0.465	0.153		0.5251	1489
	Gw-HUE		2		NCS S 2565-G			0.2081		0.4873	0.0987		0.5202	620.6
	BG-P2-2		1		NCS S 3030-B50G			0.2561		0.3415	0.1555		0.4667	856.9
	Y-HUE		1		NCS S 0580-Y			0.472		0.4445	0.2555		0.5413	1541
	YG-S3		1		NCS S 7010-G50Y			0.3196		0.3822	0.184		0.4951	262.6
	YG-T3		1		NCS S 0540-G40Y			0.3312		0.4277	0.1774		0.5153	1747
	YG-P2-1		1		NCS S 4030-G30Y			0.3234		0.4172	0.1758		0.5102	587.4
	YG-P1-1		1		NCS S 2050-G30Y			0.3168		0.4484	0.1636		0.5209	982.3
	G-T1		1		NCS S 2060-B90G			0.2073		0.4466	0.1044		0.5059	804
	G-HUE		1		NCS S 3060-B90G			0.2003		0.4425	0.1013		0.5035	606.7
	Gc-T1		1		NCS S 2555-B60G			0.199		0.3798	0.1112		0.4774	722.3
bùlúù	Bw-HUE		4		NCS S 4050-R80B			0.1977		0.1861	0.1635		0.3462	252.2
	B-S1		3		NCS S 4050-R90B			0.1949		0.2255	0.1467		0.3818	285.8
	G-HUE		2		NCS S 3060-B90G			0.2003		0.4425	0.1013		0.5035	606.7
	V-S2		2		NCS S 6020-R60B			0.2767		0.2406	0.2075		0.406	183.3
	Bc-HUE		1		NCS S 3050-B			0.187		0.2399	0.1358		0.3922	464
	BV-HUE		1		NCS S 3050-R60B			0.2431		0.2018	0.197		0.368	349.3
	BV-T1		1		NCS S 2060-R70B			0.2426		0.2106	0.1924		0.3759	432.3
	B-P1-1		1		NCS S 3050-R80B			0.2206		0.2295	0.1661		0.3887	425.7
	Bc-T1		1		NCS S 2060-B			0.1923		0.2516	0.1365		0.4019	645.9
	G-T1		1		NCS S 2060-B90G			0.2073		0.4466	0.1044		0.5059	804
	Gw-HUE		1		NCS S 2565-G			0.2081		0.4873	0.0987		0.5202	620.6
	Gw-T1		1		NCS S 1565-G			0.2257		0.4781	0.109		0.5193	894.9
	C-T1		1		NCS S 2050-B10G			0.2036		0.2892	0.1343		0.4293	771.3
	C-S2		1		NCS S 5040-B			0.2094		0.2817	0.1405		0.4252	320.1
	YO-S1		1		NCS S 3060-Y20R			0.4972		0.3861	0.2996		0.5234	670.9
	RO-T2		1		NCS S 0560-Y60R			0.513		0.3314	0.3448		0.5012	960.1
hɔ̀làhɔ̀là	YG-LT		3		NCS S 0520-G70Y			0.3457		0.3837	0.2001		0.4995	2022
	Bc-T3		2		NCS S 1050-B			0.2267		0.2965	0.1485		0.4371	1180
	O-LT		2		NCS S 0530-Y40R			0.4033		0.3532	0.2508		0.4943	1527
	Yc-T2		1		NCS S 0540-G80Y			0.3846		0.4218	0.211		0.5206	1981
	Y-S1		2		NCS S 3060-Y			0.4627		0.4205	0.2599		0.5315	790.5
	Yc-HUE		1		NCS S 0570-G80Y			0.4115		0.4803	0.2073		0.5444	1621
	YO-LT		1		NCS S 0530-Y30R			0.4011		0.3671	0.243		0.5003	1606
	Y-LT		1		NCS S 0530-Y			0.3756		0.3892	0.2171		0.5063	1926
	C-P1-2		1		NCS S 1030-B10G			0.2626		0.3193	0.1666		0.4557	1320
	M-T2		1		NCS S 2050-R30B			0.3872		0.2587	0.2906		0.4368	707.8
	G-P1-1		1		NCS S 2050-G10Y			0.263		0.4271	0.1385		0.5058	1024
	Bw-T4		1		NCS S 2040-R80B			0.2497		0.2589	0.1781		0.4155	1026
	B-P1-2		1		NCS S 2040-R80B			0.2508		0.2735	0.1736		0.4258	820.9
	BV-HUE		1		NCS S 3050-R60B			0.2431		0.2018	0.197		0.368	349.3
	BG-T4		1		NCS S 0540-B30G			0.2435		0.3375	0.1484		0.4628	1400
	BG-LT		1		NCS S 0530-B90G			0.2737		0.3528	0.1638		0.4749	1729
	BG-T2		1		NCS S 1050-B30G			0.2151		0.3363	0.1303		0.4582	1004
	O-P1-2		1		NCS S 1030-Y40R			0.4167		0.3602	0.2569		0.4996	1280
dʒùmòdʒùmò	YO-S4		2		NCS S 7010-Y30R			0.3742		0.3501	0.232		0.4883	220.1
	O-S3		2		NCS S 8005-Y50R			0.368		0.3393	0.2324		0.482	201.6
	G-S3		2		NCS S 7010-B70G			0.2736		0.3547	0.1632		0.4758	208.9
	BG-S1		2		NCS S 5030-B50G			0.2264		0.3492	0.1344		0.4664	372.9
	BV-T3		1		NCS S 1555-R70B			0.2521		0.2252	0.194		0.3899	654
	B-P2-2		1		NCS S 3030-R90B			0.2652		0.2892	0.1786		0.4382	783
	B-P1-1		1		NCS S 3050-R80B			0.2206		0.2295	0.1661		0.3887	425.7
	BG-P3-1		1		NCS S 6020-B50G			0.2588		0.3326	0.1599		0.4624	328.1
	BG-LT		1		NCS S 0530-B90G			0.2737		0.3528	0.1638		0.4749	1729
	Y-S3		1		NCS S 6020-Y10R			0.4092		0.3762	0.2445		0.5057	360.8
	G-S1		1		NCS S 4040-B90G			0.2326		0.4169	0.1235		0.4978	451.7
	YGc-T3		1		NCS S 0540-G30Y			0.3084		0.4124	0.1682		0.5062	1695
	R-S2		1		NCS S 5030-R			0.4393		0.3158	0.2972		0.4808	253.3
	M-S1		1		NCS S 4040-R20B			0.4477		0.2608	0.3421		0.4484	303.6
	O-LP		1		NCS S 1010-Y30R			0.3552		0.3463	0.2204		0.4835	1775
	BG-T3		1		NCS S 1050-B30G			0.2273		0.3359	0.1383		0.4597	1201
	G-DS		1		NCS S 8005-G20Y			0.2944		0.3573	0.1758		0.48	194.2
	Bw-HUE		1		NCS S 4050-R80B			0.1977		0.1861	0.1635		0.3462	252.2
	BG-P2-2		1		NCS S 3030-B50G			0.2561		0.3415	0.1555		0.4667	856.9
kɔ̀làkɔ̀là	GRAY 5		2		NCS S 5000-N			0.315		0.3225	0.2019		0.4651	644.9
	O-LT		2		NCS S 0530-Y40R			0.4033		0.3532	0.2508		0.4943	1527
	C-LT		1		NCS S 0520-B30G			0.2708		0.3283	0.1693		0.4618	1706
	BG-P1-1		1		NCS S 2040-B70G			0.2304		0.3615	0.134		0.4731	912.7
	V-P1-3		1		NCS S 2030-R60B			0.291		0.2758	0.2032		0.4334	973.4
	B-LT		1		NCS S 1020-R90B			0.2794		0.3089	0.1818		0.4522	1614
	M-P2-2		1		NCS S 3030-R30B			0.3647		0.2937	0.2517		0.4561	770.5
	YGw-HUE		1		NCS S 0565-G50Y			0.3677		0.5027	0.1773		0.5453	1522
	YGw-S1		1		NCS S 2060-G50Y			0.3806		0.4696	0.1933		0.5368	835.4
	GRAY 4.5		1		NCS S 5500-N			0.317		0.3242	0.2026		0.4664	594
	Y-P4-3		1		NCS S 3010-Y10R			0.3545		0.3579	0.2154		0.4891	1120
	YG-P1-2		1		NCS S 1030-G40Y			0.3281		0.3974	0.1845		0.5029	1503
	Y-T2		1		NCS S 0540-Y			0.3994		0.4136	0.223		0.5196	1860
	C-P3-1		1		NCS S 5020-B			0.2573		0.306	0.1671		0.4472	405.6
	RO-P1-2		1		NCS S 1030-Y60R			0.4076		0.334	0.2633		0.4854	1132
	Rv-LT		1		NCS S 1020-R30B			0.3361		0.2999	0.2268		0.4554	1587
	YGw-P1-1		2		NCS S 1040-G70Y			0.3681		0.4242	0.2002		0.5191	1561
	R-P1-2		1		NCS S 1030-R			0.3741		0.317	0.2471		0.4711	1277
	BG-P3-3		1		NCS S 2010-B50G			0.2932		0.33	0.184		0.466	1250
	Yw-T2		1		NCS S 0550-Y10R			0.431		0.392	0.252		0.5157	1587
tʃàràtʃàrà	YO-LT		3		NCS S 0530-Y30R			0.4011		0.3671	0.243		0.5003	1606
	RV-T3		1		NCS S 2040-R50B			0.3156		0.2556	0.2323		0.4232	871.6
	V-P2-2		1		NCS S 4020-R60B			0.2898		0.2759	0.2023		0.4333	554.6
	M-S1		1		NCS S 4040-R20B			0.4477		0.2608	0.3421		0.4484	303.6
	BV-T3		1		NCS S 1555-R70B			0.2521		0.2252	0.194		0.3899	654
	BG-S3		1		NCS S 6020-B30G			0.2371		0.3227	0.1482		0.4539	254.7
	V-P1-1		1		NCS S 4030-R60B			0.2717		0.2452	0.2013		0.4088	387.6
	V-DS		1		NCS S 7010-R50B			0.3133		0.2815	0.2179		0.4405	180.3
	BG-P1-2		1		NCS S 1030-B70G			0.2659		0.3456	0.1608		0.4702	1425
	G-P2-2		1		NCS S 3030-G10Y			0.2873		0.3737	0.1663		0.4868	962.2
	GRAY 7		1		NCS S 3000-N			0.3173		0.3246	0.2028		0.4666	1093
	M-P1-2		1		NCS S 1030-R20B			0.3607		0.2969	0.247		0.4574	1232
	YO-S1		1		NCS S 3060-Y20R			0.4972		0.3861	0.2996		0.5234	670.9
	R-P1-2		1		NCS S 1030-R			0.3741		0.317	0.2471		0.4711	1277
	YO-P1-1		1		NCS S 1030-Y30R			0.4118		0.3783	0.2453		0.507	1345
	RO-P3-1		1		NCS S 6010-Y50R			0.3812		0.332	0.2451		0.4802	388.7
	O-S2		1		NCS S 6020-Y40R			0.4367		0.3538	0.2742		0.4997	324.3
	R-P3-3		1		NCS S 2010-Y90R			0.3423		0.3216	0.2218		0.4688	1108
	C-T2		1		NCS S 1050-B10G			0.2146		0.3025	0.1384		0.4391	940.5
	M-P1-1		1		NCS S 3040-R30B			0.4004		0.2702	0.2943		0.4469	548.7
	Ø		1		no answer									
bƱ̀ɔ̀nàbƱ̀ɔ̀nà	Ø		2		no answer									
	YGw-S1		1		NCS S 2060-G50Y			0.3806		0.4696	0.1933		0.5368	835.4
	Bc-T2		1		NCS S 1060-B			0.2033		0.2682	0.1399		0.4153	870.5
	B-T4		1		NCS S 0540-B			0.2509		0.2978	0.1653		0.4414	1383
	M-S1		1		NCS S 4040-R20B			0.4477		0.2608	0.3421		0.4484	303.6
	Y-P2-1		1		NCS S 3040-Y			0.4162		0.4045	0.2371		0.5185	945.9
	B-P2-3		1		NCS S 3010-R90B			0.29		0.3063	0.1903		0.4523	1101
	V-S1		1		NCS S 5030-R60B			0.2703		0.2284	0.2079		0.3953	231.1
	RV-T2		1		NCS S 2050-R50B			0.3236		0.2463	0.2438		0.4176	648.4
	RV-LT		1		NCS S 1020-R30B			0.3361		0.2999	0.2268		0.4554	1587
	YGc-T1		1		NCS S 0565-G10Y			0.2661		0.4769	0.13		0.524	1223
	RV-T4		1		NCS S 1040-R50B			0.3209		0.2741	0.2273		0.4368	1203
	YG-P3-3		1		NCS S 2020-G50Y			0.3331		0.3678	0.1974		0.4906	1284
	YG-S1		1		NCS S 4050-G30Y			0.2965		0.4815	0.1449		0.5295	475.8
	C-T4		1		NCS S 1040-B20G			0.2462		0.3231	0.1542		0.4554	1436
	YG-P1-2		1		NCS S 1030-G40Y			0.3281		0.3974	0.1845		0.5029	1503
	BG-LT		1		NCS S 0530-B90G			0.2737		0.3528	0.1638		0.4749	1729
	V-P2-1		1		NCS S 5020-R60B			0.2886		0.2744	0.202		0.4321	330.3
	O-LP		1		NCS S 1010-Y30R			0.3552		0.3463	0.2204		0.4835	1775
	YGw-P2-1		1		NCS S 3040-G80Y			0.3799		0.4386	0.2025		0.5261	909.7
	B-T3		1		NCS S 4030-R60B			0.2312		0.2782	0.1574		0.4261	1100
	Y-P1-1		1		NCS S 1040-Y			0.4079		0.4121	0.2288		0.5202	1503
ʔìlèʔìlè	GRAY 2		2		NCS S 7500-N			0.3163		0.3226	0.2028		0.4654	217.6
	YG-HUE		1		NCS S 0575-G20Y			0.2926		0.5276	0.1338		0.5429	1173
	BG-S1		1		NCS S 5030-B50G			0.2264		0.3492	0.1344		0.4664	372.9
	V-P2-3		1		NCS S 3020-R60B			0.3003		0.2947	0.2024		0.4468	971.8
	O-S3		1		NCS S 8005-Y50R			0.368		0.3393	0.2324		0.482	201.6
	BG-HUE		1		NCS S 3050-B30G			0.1952		0.3261	0.1197		0.45	543.3
	Y-DS		1		NCS S 8005-Y20R			0.3441		0.3455	0.2131		0.4815	170.1
	G-S2		1		NCS S 6020-B90G			0.2513		0.3904	0.1399		0.4892	282.7
	BV-T2		1		NCS S 2060-R70B			0.2393		0.2048	0.1923		0.3702	561.7
	BLACK		1		NCS S 8500-N			0.32		0.3255	0.2043		0.4675	90.29
	V-S2		1		NCS S 6020-R60B			0.2767		0.2406	0.2075		0.406	183.3
	YGw-S4		1		NCS S 7010-G90Y			0.3586		0.366	0.2149		0.4935	242.8
	G-T1		1		NCS S 2060-B90G			0.2073		0.4466	0.1044		0.5059	804
	YGc-T2		1		NCS S 0550-G20Y			0.2927		0.4441	0.1512		0.5161	1523
	GRAY 6		1		NCS S 4000-N			0.3181		0.3256	0.2029		0.4673	887.9
	B-HUE		1		NCS S 3060-R90B			0.1903		0.2109	0.1478		0.3685	351.2
	R-LP		1		NCS S 1010-Y90R			0.343		0.3234	0.2215		0.4699	1659
	RO-S3		1		NCS S 7010-Y70R			0.3784		0.3331	0.2425		0.4804	210.2
	C-S2		1		NCS S 5040-B			0.2094		0.2817	0.1405		0.4252	320.1
	G-S3		1		NCS S 7010-B70G			0.2736		0.3547	0.1632		0.4758	208.9
	B-T1		1		NCS S 2060-R90B			0.1937		0.2337	0.143		0.3883	632.6
	BG-P1-2		1		NCS S 1030-B70G			0.2659		0.3456	0.1608		0.4702	1425
Bùsàbùsà	YO-P1-1		2		NCS S 1030-Y30R			0.4118		0.3783	0.2453		0.507	1345
	Y-P1-1		1		NCS S 1040-Y			0.4079		0.4121	0.2288		0.5202	1503
	BV-T5		1		NCS S 1040-R70B			0.2752		0.2656	0.1953		0.424	1073
	Bw-T5		1		NCS S 1040-R80B			0.2668		0.2792	0.1834		0.432	1234
	B-P2-1		1		NCS S 5030-R90B			0.2492		0.2715	0.173		0.4242	342.2
	YG-P2-3		1		NCS S 1030-G40Y			0.3292		0.3832	0.1897		0.4969	1370
	B-P1-1		1		NCS S 3050-R80B			0.2206		0.2295	0.1661		0.3887	425.7
	V-S2		1		NCS S 6020-R60B			0.2767		0.2406	0.2075		0.406	183.3
	Gc-T2		1		NCS S 2050-B70G			0.2083		0.384	0.1158		0.4806	962.6
	YGw-S2		1		NCS S 4040-G70Y			0.3841		0.4355	0.206		0.5256	506.2
	B-LT		1		NCS S 1020-R90B			0.2794		0.3089	0.1818		0.4522	1614
	Y-S1		1		NCS S 3060-Y			0.4627		0.4205	0.2599		0.5315	790.5
	G-P3-1		1		NCS S 6010-B90G			0.2863		0.3561	0.1709		0.4783	410.6
	Gc-T1		1		NCS S 2555-B60G			0.199		0.3798	0.1112		0.4774	722.3
	C-P3-3		1		NCS S 2010-B			0.2914		0.3212	0.1859		0.4609	1240
	G-S2		1		NCS S 6020-B90G			0.2513		0.3904	0.1399		0.4892	282.7
	YO-LT		1		NCS S 0530-Y30R			0.4011		0.3671	0.243		0.5003	1606
	O-LP		1		NCS S 1010-Y30R			0.3552		0.3463	0.2204		0.4835	1775
	Y-LT		1		NCS S 0530-Y			0.3756		0.3892	0.2171		0.5063	1926
	Y-LP		1		NCS S 1015-Y20R			0.3616		0.3625	0.2183		0.4923	1806
	C-P1-2		1		NCS S 1030-B10G			0.2626		0.3193	0.1666		0.4557	1320
	YO-P2-2		1		NCS S 2030-Y20R			0.4014		0.3727	0.2407		0.5029	1280
		*x*		*y*		*u*′	*v*′		Luminance (cd/m^2^)		
Reference white sample		0.3159		0.3218		0.2028	0.4649		2421		

*Note.* NCS = Natural Colour System.

  ‘Of all these things, which one is really *asIama*?’

Using a short baton, the consultants were instructed to point at a single colour tile that they believed best fit the given term. The experiment did not check the range of each term, only the best example for all colour terms presented. While no tests for colour vision were carried out, typical results of red–green colour blindness do not take part in the match.

The 314 colour tiles measured 0.5 × 0.8 inches each and were distributed over four charts (81/2 × 11 inches each; Color-aid Corporation, 1989–2003). The ‘Extra Hues’ section of Chart 1 was deliberately covered as it contained 10 tiles (i.e., R, Y, B, G, YO, Yc, Gw, BG, Rc and RO) at their highest point of saturation, which may have affected the consultants’ point of gaze and attracted too much of their attention; thus, the four charts contained a total of 304 colour tiles. The goal was to encourage the consultants to visually search through all four charts until they identified the best example.

Based on the results from the tile-naming task, the following groups were isolated for the focal task: (a) dominant, (b) name-an-object (i.e., the name of an object that is characteristically of the corresponding colour; see Berlin & Kay, 1969; e.g., *sƱ́sáƱ̀* refers to the flour of the dawadawa fruit and *sƱ̀ɔ̀sɛ́nÍÍ* refers to the broth made from boiled bean leaves), (c) borrowed and (d) ideophones. Table 1 displays the colour terms belonging to each group.

**Table 1.** Colour Categories.

Category	Colour terms
Dominant	pƱ̀mmá, búmmò, sÌàmá
Name-an-object	sƱ́sáƱ̀, sƱ̀ɔ̀sɛ́nÍÍ
Borrowed	bùlúù
Ideophones	hɔlàhɔlà, bƱ̀ɔnàbƱ̀ɔnà, bùsàbùsà, dʒùmòdʒùmò, tʃàràtʃàrà, kɔlàkɔlà, ʔìlèʔìlè

## Colour-Mapping Method and Representation

A new method was introduced to determine the CIE values for the full set of 314 Color-aid tiles using photometrical measurements. One of the goals of colour science is to specify the quality of colour using certain colour spaces. Some colour spaces separate the three dimensions of colour into one luminance dimension and two chromaticity dimensions while also maintaining perceptual uniformity (e.g., the CIELAB model). Within a colour space, which is considered perceptually uniform, a unit change in colour value should produce a similar change in visual importance, independent from the location.

Colour values can be transformed into a graphical diagram, known as the CIE (*x, y*) chromaticity diagram, which provides a two-dimensional representation. However, this diagram does not have a direct perceptual correlation; hence, many different chromaticity diagrams have been proposed by squeezing or stretching the CIE (*x, y*) chromaticity diagram so that it better represents the correlations between perceived colour differences and the corresponding distances in the diagram. The *x* and *y* variables were transformed into a new set of values (*u, v*) through a set of mathematical formulas (DeCusatis, 1997). In 1976, the CIE standardised the (*u′, v*′) diagram as an approximately uniform chromaticity diagram. This was achieved by multiplying the values for *v* by 1.5; thus, in the new diagram, *u*′ = u and *v*′ = 1.5v.

**Figure 2.**
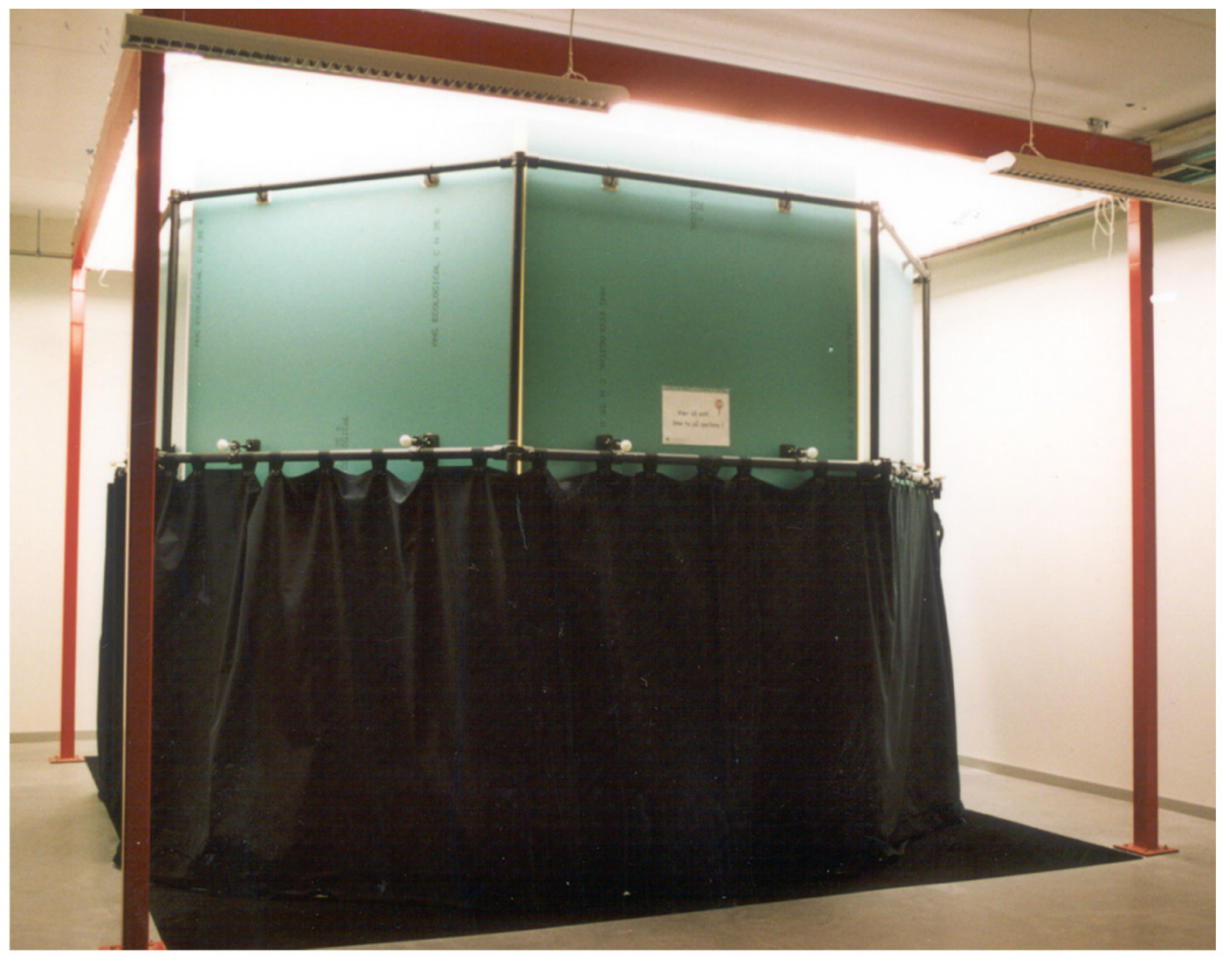
The artificial sky room.

The values of *u*′ and* v*′, as well as other photometrical values, in the whole Color-aid set were measured under an artificial sky, which simulates overcast (i.e., creates even and diffused illumination; see B. Matusiak & Arnesen, 2005; B. S. Matusiak & Braczkowski, 2014; Figure 2), at the Daylight Laboratory of the Department of Architecture and Technology, Light & Colour Centre, NTNU. The ceiling of the artificial sky room is covered in RGBW LED-chips, which can generate light with different correlated-colour-temperature (CCT) values.

In this study, a CCT of 6,500K was used. To provide stable and comfortable illumination, the illuminance level under the artificial sky was adjusted to 50% of the maximum power, imparting an illuminance of around 8,610 lux onto the Color-aid tiles. The tiles were successively placed on a table located in the middle of the artificial sky room. The photometrical measurements were taken using the SpectraScan PR-655 tool (PhotoResearch) to determine the CIE coordinates for all 314 colour tiles. Each tile had a matte finish and measured 2 × 3 inches.

**Figure 3.**
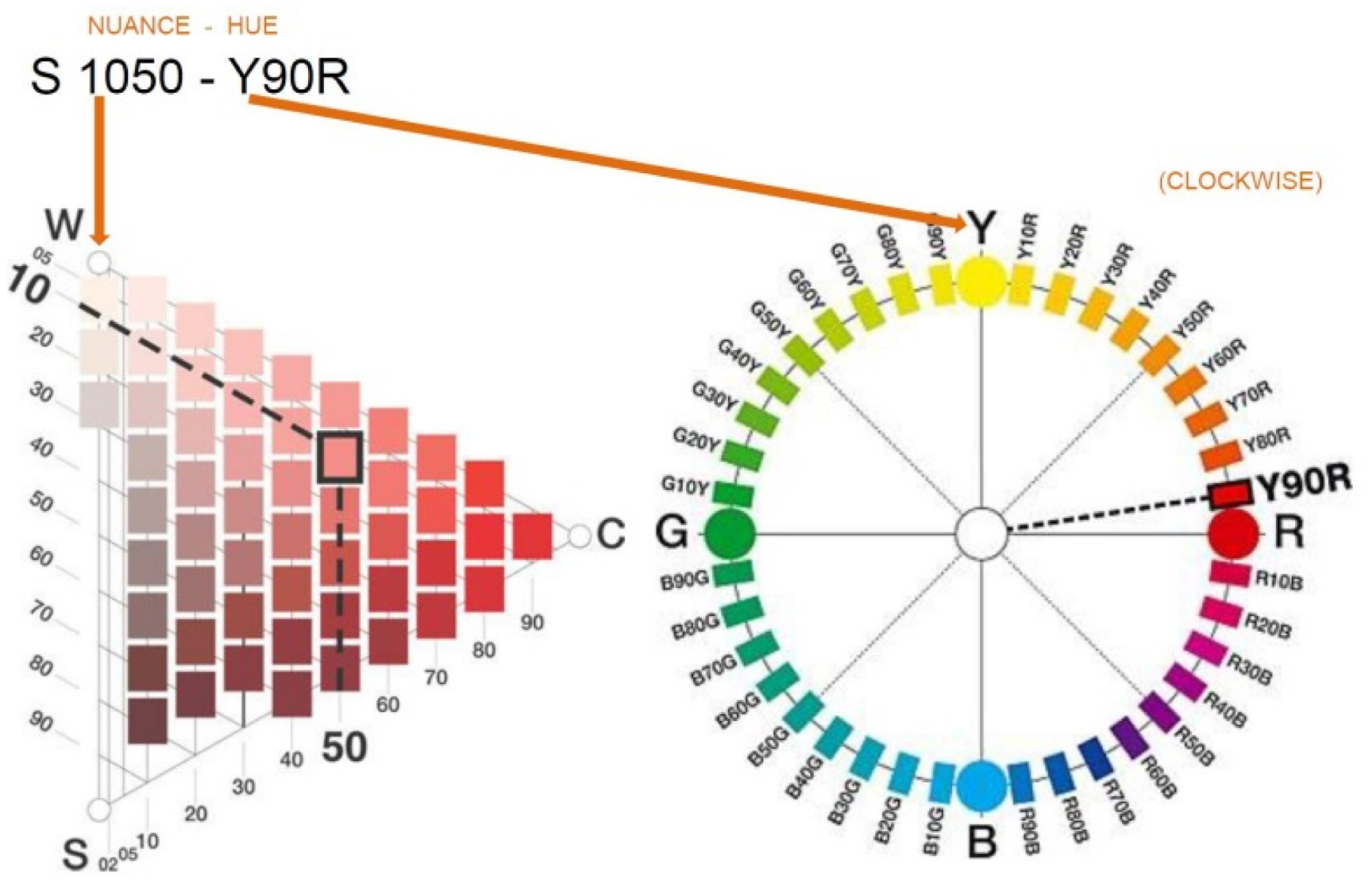
NCS colour system.

As indicated earlier, the CIE system is not easily interpreted. Since the NCS provides a graphical representation of colours that is more easily understood, both by professionals and laypeople, we decided to use the NCS to represent the results of this study, in addition to the CIE (*u′, v*′) diagram. The NCS uses a numeric code that describes both the hue and nuance of each colour (Hård & Sivik, 1981). For example, S1050-Y90R describes a colour that includes yellow with 90% perceived red in hue, with a nuance of 10% blackness and 50% chromaticness (Figure 3). We used the NCS Colour Scan tool to find the NCS code corresponding to each Color-aid tile, finding that some of the Color-aid tiles may share the same hue but have different nuances, and vice versa. The NCS codes are presented in Table 2.

## Results

The result of the focal task in Brindle (2016) indicates that a wide range of colour tiles were assigned to each ideophonic expression, which suggests that the selected ideophonic expressions cannot be reliably ascribed to a recognisable colour region. Converting the 314 Color-aid tiles into the CIE numerical values allowed us the opportunity to diagnose Brindle’s data and answer the questions raised. This section proposes how one can approach situations in which ideophones cannot be said to denote ‘colours’, as categorised by the Color-aid Corporation.

In Table 2, the Chakali expressions, the Color-aid tile code, the number of consultants who pointed at each tile, the NCS code, the *x*-value, the *y*-value, the *u*′-value, the *v*′-value and the luminance (cd/m^2^) are aligned from left to right. It can be observed that the terms *pƱ̀mmá*, *búmmò* and *sÌàmá* are, respectively, clustered around the whitish-light, blackish-dark, and reddish tiles. The name-an-object terms are predominantly associated with specific regions: *sƱ́sáƱ̀* ‘dawadawa flour’ with the yellowish and orangish tiles, and *sƱ̀ɔ̀sɛ́nÍÍ* ‘bean-leaf water/broth’ with the greenish tiles. The English loanword, *bùlúù*, clearly indicates a ‘grue’ category, as the consultants mainly identified bluish and greenish tiles as best fits for the term. Only two unusual responses produced results in the yellow-brownish (YO-S1) and soft red-beige (RO-T2) areas. For the ideophones, the consultants identified tiles that are distributed across the charts and, therefore, cannot be reliably ascribed to a familiar colour space or category.

The lists of tile codes in Table 2 show that all ideophones can be represented with warm and cool hues. Some could be delineated with negative statements; for example, *ʔileʔile* was assigned to 22 tiles without redness (R). Regardless, we argue that there are no defining properties among the ideophones; thus, the only generalisation that can be made is that no colour categorisation emerged in this category. There were almost as many tiles identified for each ideophone as there were consultants, leading us to believe that these ideophones may not have foci in the Ostwald or other colour systems, with perhaps one exception: *hɔ̀làhɔ̀là*. This term was assigned to the T (tint) and LT (light tint) tiles during the focal task, which may indicate that *hɔ̀làhɔ̀là* can be defined as a ‘pale variation of any hue’ or ‘pastel’. We return to this problem in the ‘Statistical analysis’ and ‘Discussion and conclusion’ sections.

Results from the focal task provide evidence that *búmmò*,* pƱ̀mmá* and *sÌàmá* are defined by the most centrally concentrated colour terms, followed by *sƱ́sáƱ̀*, *sƱ̀ɔ̀sɛ́nÍÍ* and bùlúù, while the ideophones are not denoted by the ‘colours’ categorised by the Color-aid Corporation; however, but could possibly be of the ‘desaturated colour-type’ (MacLaury, 2007).

**Figure 4.**
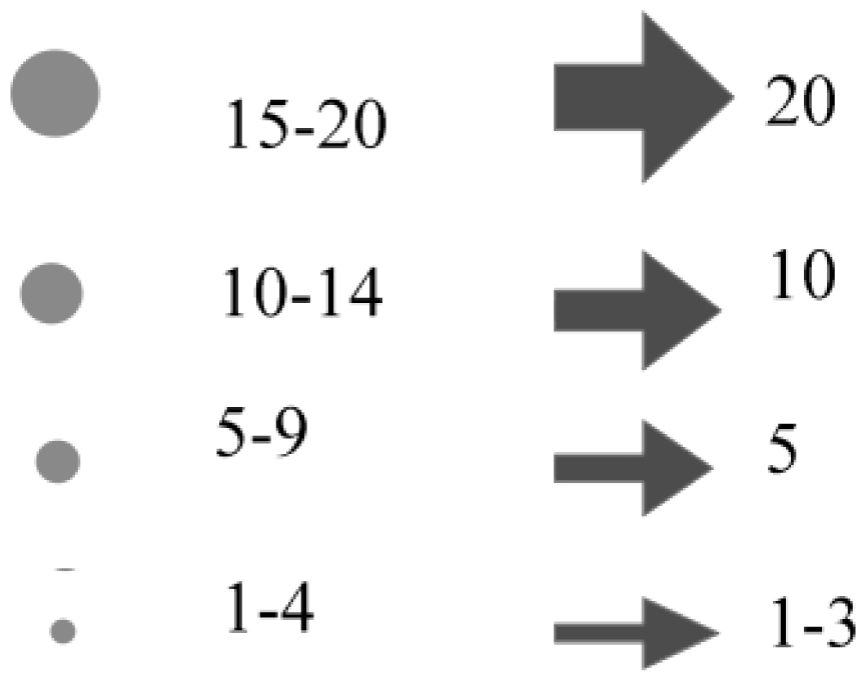
Graphics used to represent the number of subjects.

**Figure 5.**
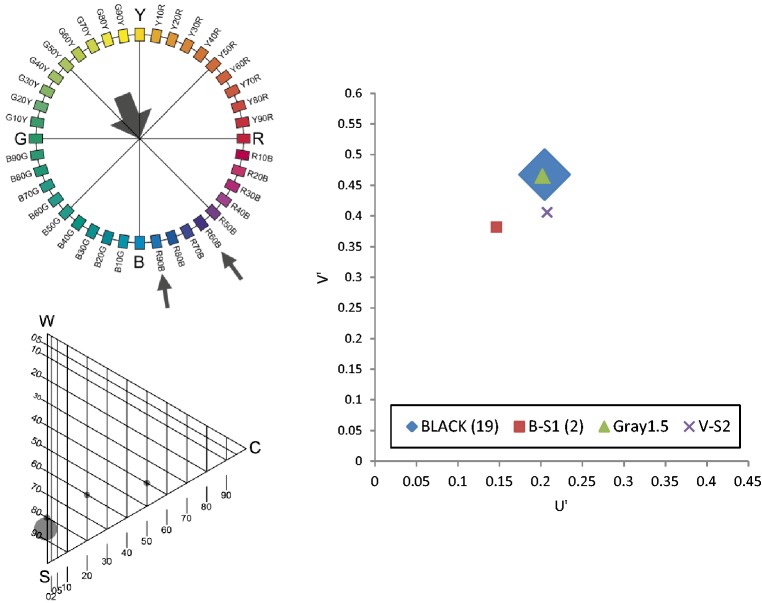
Example diagrams for Category 1: búmmò.

**Figure 6.**
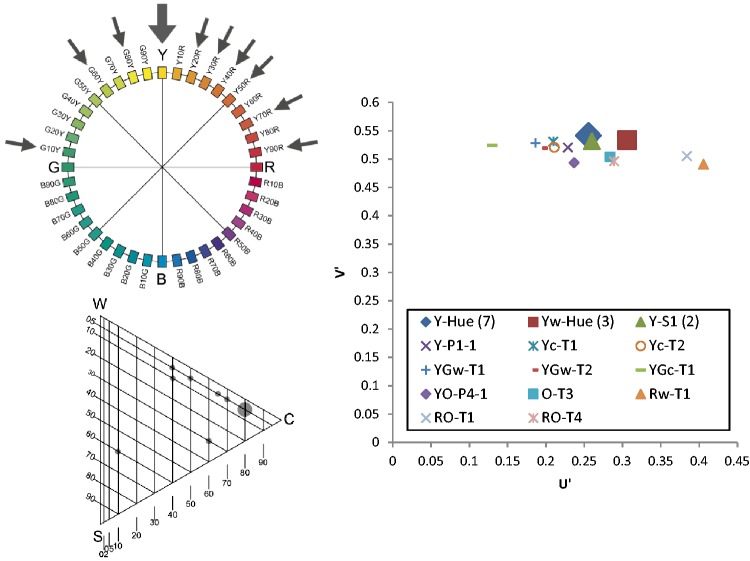
Example diagrams for Category 2: sƱ́sáƱ̀.

**Figure 7.**
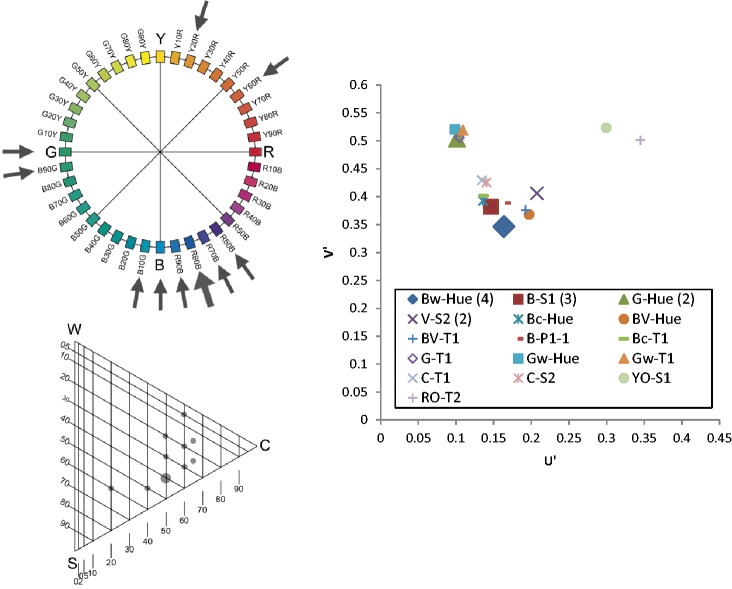
Example diagrams for Category 3: bùlúù.

**Figure 8.**
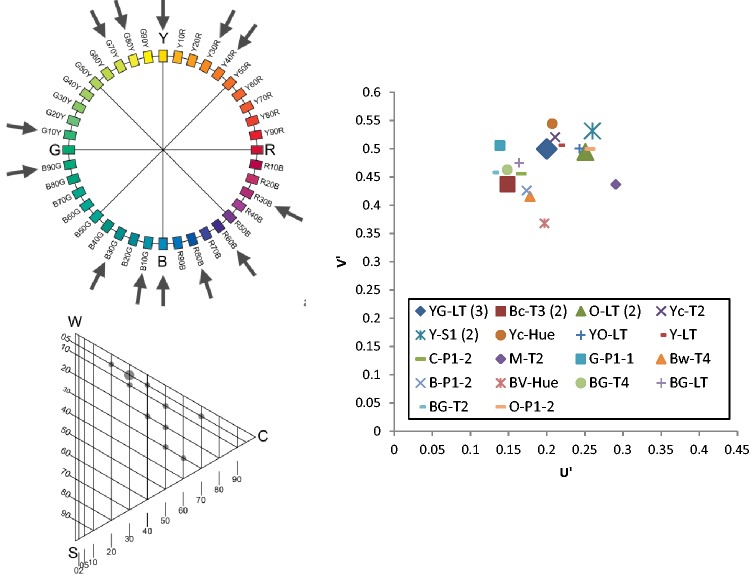
Example diagrams for Category 4: hɔlàhɔlà.

The results will be presented graphically via the NCS Colour Circle (hue), the NCS Colour Triangle (nuance) and the 1976 CIE (*u′, v*′) chromaticity diagram. To simplify the presentation, the graphics in Figure 4 were used. One example from each category was presented via the NCS circle and triangle diagrams, as shown in Figures 5 to 8. The CIE (*u′, v*′) chromaticity and spectral power distributions were measured for all colour terms and are available from the authors upon request.

Based on results presented in Table 2 and Figures 5 to 8, we can conclude the following:

1. The term *búmmò*, as presented in Figure 5, may be an appropriate expression for ‘dark-grey’ or ‘near-black’ since most of the consultants (20 out of 23) selected a grey tile with 85% blackness.
2. The term *sƱ́sáƱ̀*, as presented in Figure 6, may be an appropriate expression for a yellowish colour, but with a wide range of yellow hues with different nuances. Most of the nuances have the same level of blackness, which is 5%.
3. The term *bùlúù*, as presented in Figure 7, may describe a bluish colour but one that is unclear in hue and nuances.
4. The term *hɔ̀làhɔ̀là*, as presented in Figure 8, does not refer to any specific colour as the assigned tiles are distributed across the NCS Colour Circle. The nuances are also very different and do not have a clear pattern. We can propose that the selected colours are more pale (have more whitish).

### Statistical Analysis

A statistical analysis was also carried out. To calculate the similarities between tiles selected by the consultants based on their perceptions of the colour terms, the data were split into two categories: hue and degree of blackness.

**Figure 9.**
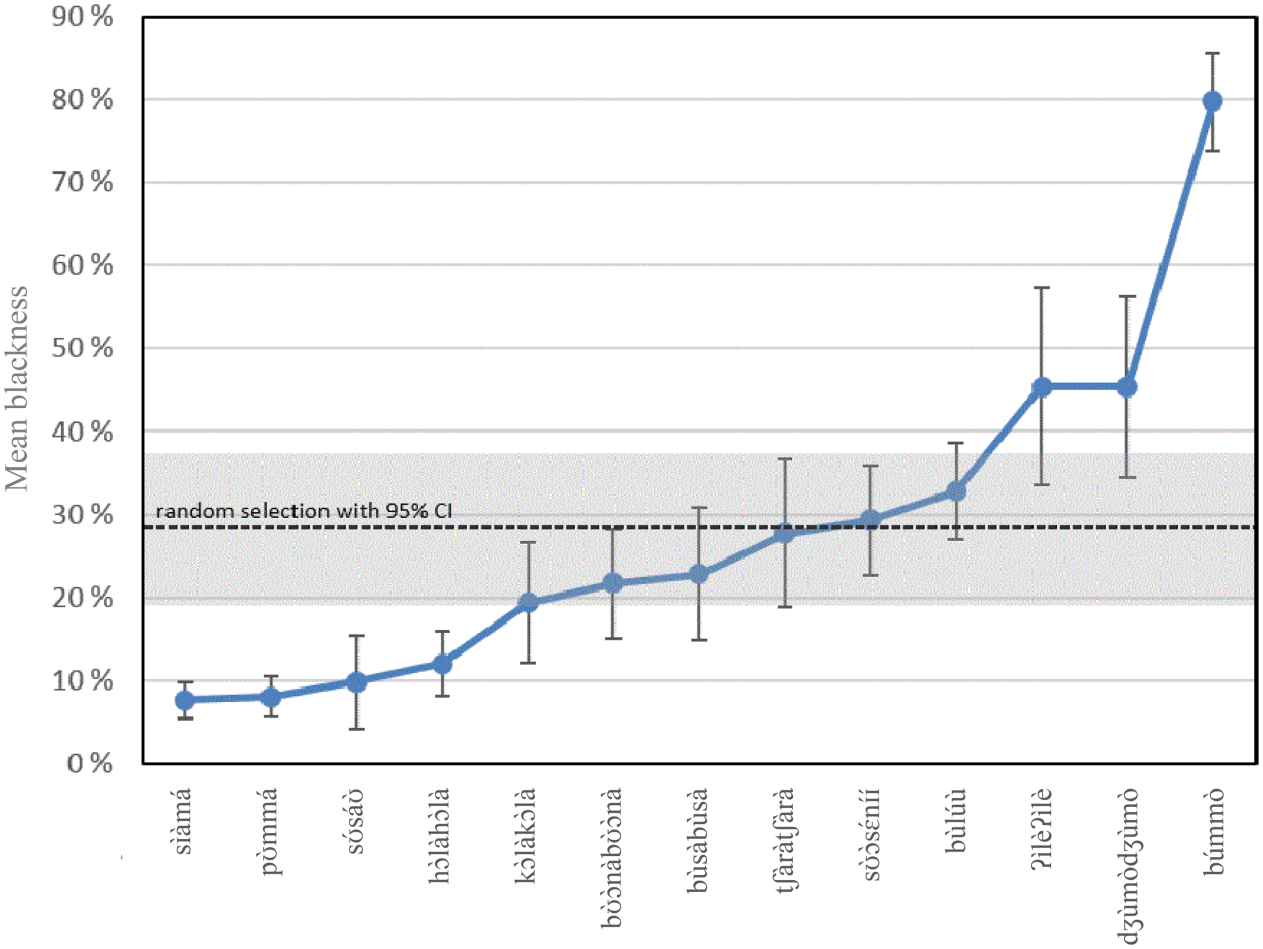
Mean degree of blackness and the 95% CIs of the colour tiles selected for each term. Note: The dotted line indicates the mean degree of blackness resulting from the 50 random draws, including its mean 95% CI. It can be observed that *hɔ̀làhɔ̀là* has the third smallest 95% CI in degree of blackness.

Figure 9 displays the mean degree of blackness among the selected colour tiles for the studied colour terms with the 95% confidence interval (CI). The 95% CI represents the degree of certainty for the mean degree of blackness, which, in turn, depends on the standard deviation. The 95% CI also represents the degree of agreement among the consultants: the smaller the CI, the greater the degree of agreement. The strongest agreement on the degree of blackness can be found in the colours selected for *sÌàmá* and *pƱ̀mmá*, while the strongest disagreement can be found in the colours selected for *ʔìlèʔìlè* and *dʒùmòdʒùmò*.

For the hues, figuring out the degree of similarity is not as straightforward. We selected the NCS Colour System to achieve this, since the hues are arranged in a circle and distances between the different hues can be calculated using the degrees of the circle. We thus translated every hue of the colour circle into a degree, with 9° marking the distance between two neighbouring hues. Then, for each pair of colour tiles selected (i.e., answers given by two consultants) for the same colour term, the shortest distance in the circle was calculated in degrees. The distances were then averaged across all possible pairs to indicate the average disagreement. For *búmmò* and *pƱ̀mmá*, no mean distance in hue could be calculated because most consultants chose a tile that did not include hue, only different degrees of blackness.

To test if these calculations are significantly different from those resulting from a random draw of the colour tiles, we ran 50 simulations in which 23 colour tiles were randomly drawn from the total array of 304 tiles with replacement. We then calculated the average degree of the mean blackness among the 50 samples (represented by the dotted line in Figure 9) as well as the mean of the 95% CIs (represented by the grey box in Figure 9). The four leftmost colour terms in Figure 9 were assigned colour tiles with a significantly lower degree of blackness than those randomly drawn from the full array (i.e., the CI for these four terms do not overlap with the randomly drawn 95% CIs), and only *búmmò* has a significantly higher degree of blackness.

**Figure 10.**
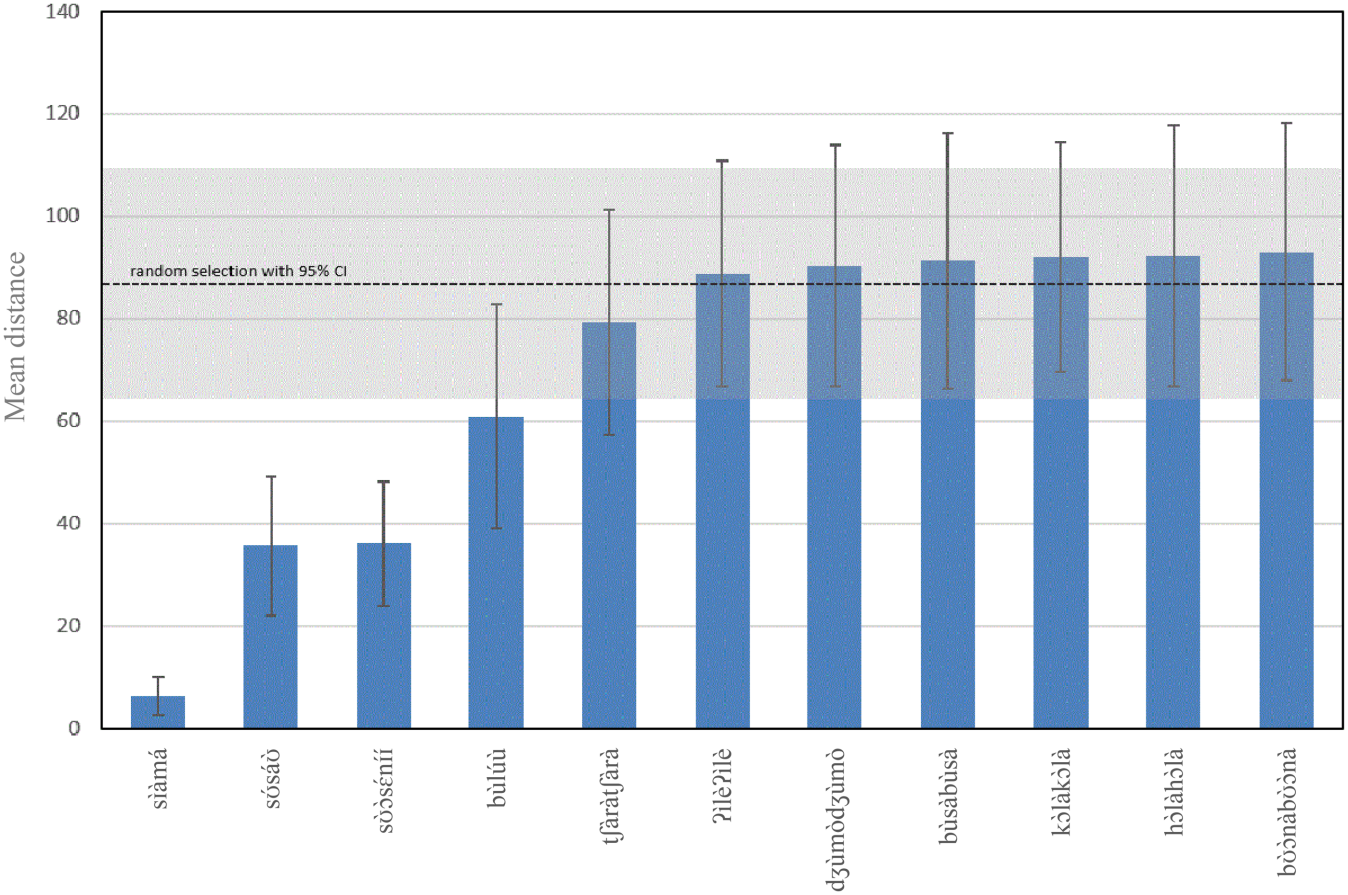
Average dissimilarity between the colour tiles selected for each category and the 95% CIs. Note that the dotted line indicates the mean distance resulting from the 50 random draws, including its mean 95% CI.

Figure 10 displays the mean distance in degrees on the NCS Colour Circle between the selected colour tiles for each term, along with their 95% CIs. To test if these calculations are significantly different from those resulting from a random draw of the colour tiles, we ran 50 simulations in which 23 colour tiles were randomly drawn from the total array of 304 tiles with replacement. We then calculated the average distances between the colour tiles in all 50 samples (represented by the dotted line in Figure 10) as well as the mean 95% CIs (represented by the grey box in Figure 10). The colour tiles selected for the three leftmost colour terms have significantly lower distance than those randomly drawn from the colour samples (i.e., the CIs for these three terms do not overlap with the randomly drawn 95% CIs), which means that there is a significantly greater degree of agreement among the consultants regarding the colours tiles selected for these terms, compared to what would be expected if they chose the tiles at random. For all the other terms, however, it cannot be rejected as the patterns resulted from a random selection of colour.

## Discussion and Conclusion

This article presented the photometrical measurements (colour coordinates) for 314 Color-aid tiles made in the Daylight Laboratory at the NTNU. The developed method offers value beyond this study and every researcher is permitted to use the dataset, which is provided in Online Appendix. The article began by presenting the original measurements of all the tiles in the Color-aid Corporation Chart, then narrowing down the discussion to the focal task experiment.

In the focal task, the goal was to understand the meaning of Chakali colour terms in terms of established colour systems. The results were obtained with the help of the Color-aid system, the NCS Colour Circle, the NCS Colour Triangle and the 1976 CIE (*u′, v*′) chromaticity diagram. The CIE coordinates and the NCS codes were calculated using the SpectraScan and NCS colour scanner tools, respectively. The range of tiles selected for each colour term was analysed in terms of their *u*′ and *v*′ values (CIE) and their hues and nuances (NCS).

Several studies have demonstrated that speakers of different languages may segment the colour spectrum differently and that some nonchromatic properties may serve as variables to determine colour categories (Biggam, 2012; Levinson, 2000; MacLaury, 2007). For example, Conklin’s (1955) study on Hanunoo colour terms showed that the variable of wetness or dryness could override hue when establishing the appropriate colour term.

Based on the visual inspections, the data of this study suggest that the best example could not be found for some terms, as the degree of variation is too significant to identify a particular region of the colour space. On the face of it, there is no consensus and an intermediate range is not easily conceived.

Before eliminating the chromatic properties and testing for various determinants, the measurements of the tiles were converted into CIE coordinates to expose emerging clusters that could be deduced from their chromatic properties, such as light/pale versus dark, or warm versus cool.

If it turns out to be impossible to map the colour terms onto the Color-aid Chart, the remaining questions are: What may account for that impossibility and how could we test it?

This article concludes with some remarks on the potential applications for the measurements and the colour perception vocabulary of Chakali. We found that most of the Chakali terms were not clear enough for the consultants to select or label them with the related colour tiles, which suggests that naming and linguistic categorisations may affect recognition. However, the findings were particularly interesting for the ideophone *hɔ̀làhɔ̀là*, which might have some characteristics of colour. This term is highly frequent, but the consultants could not agree on where the colour should be mapped. Regarding hue, the consultants’ answers spread across the NCS Colour Circle, while their answers on the NCS Colour Triangle are found rather close to pure white and can therefore be interpreted as having whitish nuances.

The statistical analysis revealed that among the colours selected for *hɔ̀làhɔ̀là* or *hɔ̀hɔ̀là*, similar to other ideophones included in this study, the mean distance between any two hues (dissimilarity) was about 90 (i.e., 90° on the NCS Colour Circle), which is also the distance between two consecutive primary colours (e.g., pure yellow and pure red, pure red and pure blue, etc.). In addition, contrary to other ideophones, *hɔ̀làhɔ̀là* or *hɔ̀hɔ̀là* refers to a colour with a very low degree of blackness (i.e., between 5% and 20%, with a mean degree of blackness of about 12%). For the rest of the ideophones, it was not easy to predict which colour they referred to.

The primary meaning of the remaining ideophones must be further investigated. In this study, we tried to categorise them but did not achieve with any convincing results. Therefore, we posit that these ideophones do not represent colours, but they may be connected to visual perception in some manner.

We propose that the developed method, consisting of the CIE and NCS systems and supplemented by a novel approach to statistical analysis, could be a very useful tool for gaining a better understanding of how precise different languages are in terms of colour naming, even when there are no equivocal colour terms in English. This method enables a precise, numerical estimate of the dissimilarity between the colours selected, both in terms of blackness and hue.

## Supplemental Material

appendix updated version 1 -Supplemental material for Categorisation of Colour Terms Using New Validation Tools: A Case Study and Implications Click here for additional data file. Supplemental material, appendix updated version 1 for Categorisation of Colour Terms Using New Validation Tools: A Case Study and Implications by Shabnam Arbab, Jonathan A. Brindle, Barbara S. Matusiak and Christian A. Klöckner in i-Perception

## Notes

1 The rationale behind selecting 65 colour tiles from the standard set (220) was described by (I. R. L. Davies et al., 1992).

2 This method was inspired by (I. Davies & Corbett, 1994, p. 79)

3 See (Childs, 1994; Dumestre, 1998).

4 The interested reader may also consult http://ideophone.org/working-definition and http://ideophone.org/three-misconceptions-about-ideophones/#footnote_0_103
